# Pentaerythritol and Glycerol Ester-Based Rosin-Modified Hydroxyl-Terminated Polybutadiene (HTPB)

**DOI:** 10.1021/acspolymersau.4c00089

**Published:** 2025-01-15

**Authors:** Frank Lee, Aran Guner, Ken Lewtas, Tony McNally

**Affiliations:** † International Institute for Nanocomposites Manufacturing (IINM), WMG, University of Warwick, WarwickCV4 7AL, U.K.; ‡ The Falcon Project Ltd., Astley, Manchester M29 7NW, U.K.

**Keywords:** pentaerythritol ester of rosin, glycerol ester of rosin, HTPB, binders, mechanical properties

## Abstract

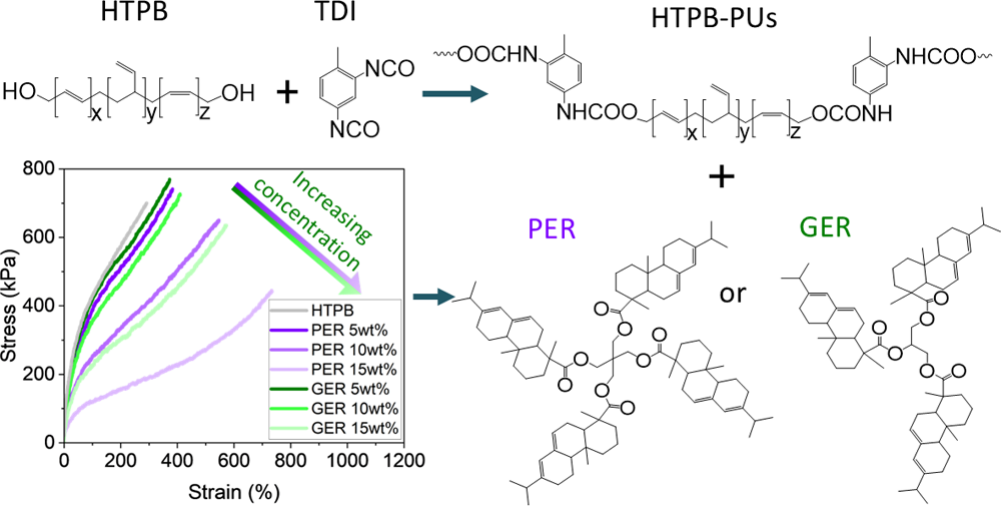

Hydroxyl-terminated polybutadiene (HTPB) has widespread applications such as in adhesives, coatings, and solid propellants due to its durability and excellent mechanical strength when cross-linked, which can be maintained at low temperatures. However, many of the additives used to modify the properties of HTPB are not sustainably sourced nor have the functionality such that tailoring of HTPB properties can be achieved. Here, we describe the use of the pentaerythritol ester of rosin (PER) and glycerol ester of rosin (GER), sourced from gum rosin (pine trees), to modify the rheology and mechanical properties of uncross-linked and cross-linked HTPB. Both rosin esters are compatible with HTPB resulting in a change in the glass transition temperature (*T*_g_) of HTPB, which is concentration-dependent. The inclusion of PER increased the viscosity of uncross-linked HTPB more than GER due to the additional abietic functionality per molecule. The rosin esters also compete with the terminal hydroxyl groups of HTPB during cross-linking with toluene diisocyanate (HTPB-PU). Consequently, the cross-link density of HTPB-PU decreases and the molecular mass between cross-links increases with increasing PER/GER content. This competition results in a decrease in Young’s modulus and tensile strength of HTPB but a significant increase in elongation at break (+153%) and tensile toughness (+76%) of HTPB.

## 1 Introduction

Hydroxyl-terminated polybutadiene (HTPB) has aroused considerable interest in the polymer industry due to its relatively large molecular mass but low viscosity.^1,2^ This results in desirable properties; for example, HTPB-based polyurethane (PU) networks can have low surface energy, high abrasion resistance, excellent tensile strength at low temperatures, low glass transition temperature, and low electrical conductivity.^2−7^ Consequently, a variety of applications such as adhesives, coatings, and pervaporation membranes are possible.^8−12^ In particular, HTPB-based PUs are extensively applied as binders for composite solid propellants between solidified inorganic oxidizers and combustion agents owing to their stability at low temperatures and ability to bind a high solid fraction (>80%) to maximize propellant performance.^1,13−15^ In order to enhance the propellant efficiency, ongoing research has focused on optimizing mechanical and thermal properties and increasing the adhesion of HTPB-based PUs,^1,3,15^ including the incorporation of a tackifier or other agents to polymer blends. Tackifiers are widely used as pressure-sensitive adhesives (PSAs)^16−19^ to increase the adhesive energy and resist the separation of two surfaces in contact, often accompanied by a modulation of the mechanical and viscoelastic properties of the adhesives.^19−22^ While tackifiers can be either natural (e.g., rosin and their derivatives, terpenes) or synthetic (e.g., from petroleum steam-cracker feedstocks),^22,23^ the urgent need for sustainable solutions has focused the demand on naturally sourced tackifiers such as the pentaerythritol ester of rosin (PER) and glycerol ester of rosin (GER).^2^ PER and GER can be produced by the esterification of pentaerythritol (glycerol) and rosin acids, an abundant renewable natural resource from pine or conifer trees.^24,25^ While GER is usually synthesized as a single component, PER is a combination of mixtures involving the tetra- to monoesters, the chemical structures of which are given in Figure S1. Nevertheless, both rosin esters possess good compatibility with a variety of polymers and thus are widely used as tackifiers in PSAs and hot melt adhesives (HMAs).^26^ HTPB is much less used in PSAs, and there is infrequent use of tackifiers in HTPB-based PSAs.^21,27−29^ Studies tailoring the mechanical, thermal, and adhesive properties of HTPB-based PUs on addition of tackifiers are thus very limited, and essential further investigation of the effects PER and GER addition has on HTPB is required.

In this paper, we report for the first time the modification of the mechanical and viscoelastic properties of HTPB by the incorporation of PER or GER. The natural rosin derivatives are studied using Fourier-transform infrared spectroscopy (FTIR), nuclear magnetic resonance (NMR) spectroscopy, and gel permeation chromatography (GPC) and blends of HTPB and PER or GER by differential scanning calorimetry (DSC) and rheology measurements. The blends were then cross-linked using toluene diisocyanate (TDI), and the dynamic and quasi-static mechanical properties of the HTPB-PUs obtained were measured by dynamic mechanical thermal analysis (DMTA) and tensile testing. This investigation provides fundamental insights into the effect rosin esters have on HTPB networks such that tailoring the properties of HTPB-based derivatives for applications as diverse as solid propellant binders and membranes is possible.

## 2 Experimental Section

### 2.1 Materials

HTPB (SK-HTPB) was obtained from the Falcon Project Ltd., while PER (DERCOL PE 100) and GER (DERCOL GL 85) were obtained from Diamantino Malho & Co. Ltd. The chemical structures of HTPB, PER, and GER are given in Figure S1. Detailed FTIR, NMR, and GPC analysis is also presented in Section 3. Toluene diisocyanate (TDI) was obtained from Merck KGaA and used as a cross-linking agent. All materials were used as received.

### 2.2 Synthesis of HTPB/PER and HTPB/GER Blends

Respective amounts of PER at 5, 10, and 15 wt % and GER at 5, 10, 13.57, and 15 wt % loadings were dissolved in HTPB at 110 °C, followed by constant stirring for 15 min at the same temperature. Once prepared, the blends were dried under vacuum in an oven for 24 h at 60 °C. For the cross-linking reaction, TDI with a 1:1 molar ratio of hydroxyl group between TDI and HTPB was added to the HTPB/PER and HTPB/GER blends under magnetic stirring for 1 h at 60 °C. The blends were then molded (rectangular; 295 mm × 85 mm × 5 mm) and heated in an oven at 60 °C for 5 days to ensure maximum cross-linking.

### 2.3 Characterization

FTIR spectra were recorded in transmission mode using a Bruker Tensor 27 FTIR spectrometer with a resolution of 2 cm^–1^ in the range of 4000 to 500 cm^–1^; 16 scans were accumulated. ^1^H NMR measurements were carried out using a Bruker DPX-400 spectrometer and CDCl_3_ with a tetramethyl silane standard. GPC was performed using an Agilent 390-LC system under THF with 2% trimethylamine (TEA) and 0.01% butylated hydroxytoluene (BHT) with a flow rate of 1 mL min^–1^ and calibrated using poly(methyl methacrylate) standards. DSC was carried out using a Mettler Toledo DSC 1 under a nitrogen environment; pure PER and GER were heated from 0 to 130 °C at 5 °C min^–1^ to allow for complete softening, while the HTPB/PER and HTPB/GER blends were measured from −120 to 100 °C at 5 °C min^–1^. Only the second heating curve is reported here. Viscosity measurements were performed on a modular advanced rheometer system (Haake Mars III) at 25 °C with a shear rate from 0.1 to 100 s^–1^ and a 30 min preconditioning step. DMTA was performed on a Triton Tritec 2000 DMA in tensile mode at a single frequency of 1 Hz at 0.05 mm (out of 10 mm) from −105 to 100 °C at 3 °C min^–1^. 0.5% strain was within the viscoelastic region of the material as confirmed by the tensile mechanical testing as described below. Tensile mechanical testing was carried out on a Shimadzu Autograph AGS-X tensile tester using a strain rate of 50 mm min^–1^.

## 3 Results and Discussion

**Figure 1:**
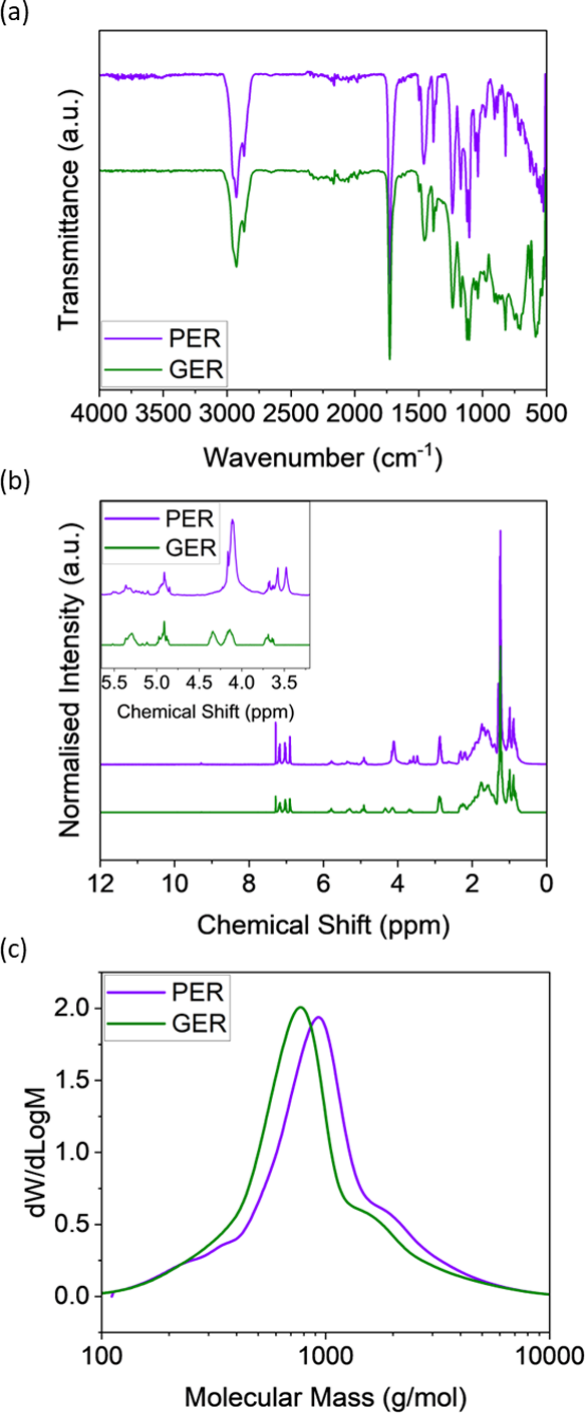
(a) FTIR spectra of pure PER and GER. (b) ^1^H NMR spectra of PER and GER. The inset shows the spectra between 3.5 and 5.5 ppm, the ester and olefinic regions for analysis of the tetraester component. (c) GPC traces of pure PER (*M*_n_: 692 g mol^–1^, PD: 1.65) and GER (*M*_n_: 639 g mol^–1^, PD: 1.67).

Figure 1a shows the FTIR spectra for both PER and GER with characteristic peaks at 2927 and 2866 cm^–1^, corresponding to the C–H stretching vibrations.^25,30^ There are strong signature bands of ester C=O stretching at 1726 cm^–1^ and C=C stretching at 1454 and 1384 cm^–1^ observed for both rosin esters. Overall, both PER and GER display similar characteristic peaks in the infrared, as reported in previous studies,^30,31^ except that PER also displays additional low-intensity peaks around 3512 cm^–1^, associated with alcohol O–H stretching, suggesting a considerable amount of unreacted hydroxyl group from pentaerythritol during esterification.^32^ The unreacted hydroxyl group(s) in PER arise due to steric hindrance,^25,32^ and in turn, PER is a combination of tetraester, triester, diester, and monoester species, the quantification of which can be evaluated using NMR and GPC, as described in the following section.

The ^1^H NMR spectra of PER and GER are shown in Figure 1b. The absence of peaks between 9 and 11 ppm indicates the absence of carboxylic acid, while peaks at 7–7.5 ppm are attributed to the aromatic functionalities of the abietic group, the primary component of resin.^25,33^ All possible rosin structures have been described previously by Lewtas et al.^34^ Focusing on the chemical shift between 3.3 and 5.5 ppm (Figure 1b, inset), which represents the ester and olefinic groups, for PER, the strong peak at 4.11 ppm corresponds to −CH_2_–O from the ester, while peaks at 3.48, 3.58, and 3.67 ppm are related to −CH_2_–OH from the alcohol. The calculation of the ratio of the area of these two regions enables an evaluation of the tetraester composition (see Figure S1), and the mole fractions of tetra-, tri-, di-, and monoesters were found to be 47.2, 39.6, 13.1, and 0.0%, respectively. The abundance of hydroxyl groups in PER may lead to side reactions with TDI during cross-linking, which will be discussed in later sections.

The evaluation of the tetraester component is also possible by GPC (Figure 1c). The number-average molecular mass of PER (692 g mol^–1^) is higher than that of GER (639 g mol^–1^) due to an extra abietic group per molecule. Nevertheless, as it seems the molecular mass ratio is significantly smaller than the 4:3 ratio as predicted from their chemical structure (4 abietic groups in PER and 3 in GER) and hidden peaks in the GPC trace of PER, it can be speculated that PER is composed of a distribution of tetra-, tri-, di-, and monoesters. Upon peak deconvolution analysis (see Figure S2), the mole fractions were determined to be 55, 30, 15, and 0% respectively, in good agreement with the NMR data. The above characterization provides fundamental information when studying the interaction of PER and GER with HTPB.

**Figure 2:**
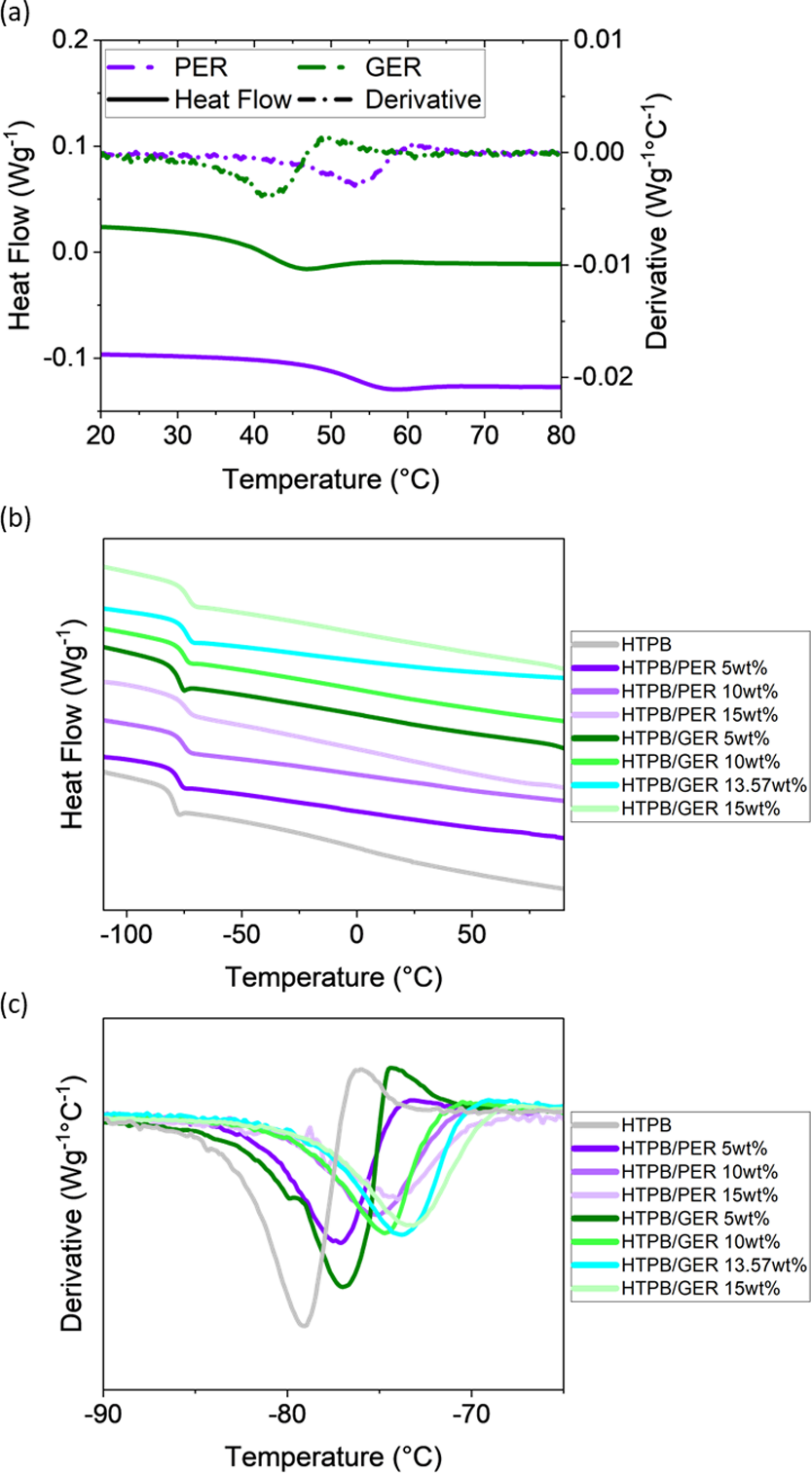
(a) DSC curves (solid lines) of pure PER and GER. Their respective slopes are shown as dash-dotted lines. (b) DSC curves (second heating). (c) Slopes at the inflection point for the HTPB/PER and HTPB/GER blends. An increase in the PER or GER concentration results in an increase in the *T*_g_.

To understand the performance and the service temperature application of blends of HTPB and PER or GER, it is essential to identify the variation in the glass transition temperature (*T*_g_) of HTPB as a function of PER and GER concentration. In the first instance, DSC was performed on pure PER and GER (Figure 2a), and after the extrapolation of the inflection point using the corresponding derivative curves, the *T*_g_ values of PER and GER were determined to be 53 and 42 °C, respectively. PER has a higher *T*_g_ than GER due to its additional abietic group, leading to a reduction in free volume.^30^ Blends of HTPB and PER or GER for loadings of 5, 10, and 15 wt % were then prepared, and the viscoelastic properties of all the blends were studied. Additionally, a blend of HTPB with 13.57 wt % GER was also prepared for comparison to the 15 wt % HTPB/PER blend, given their equivalent mole fraction, from GPC results. The above blends were then analyzed by DSC, where the heat flow traces and the corresponding slopes at the inflection are given in Figure 2b,c, respectively. The presence of a single *T*_g_ at low temperatures near that for HTPB and the disappearance of the *T*_g_ of the rosin esters are indicative of a high degree of miscibility between the blend components. *T*_g_ increases with the rosin ester concentration as predicted by the Gordon–Taylor equation,^35^ where the *T*_g_ of a homogeneous blend of two polymers can be related to its constituent polymers from
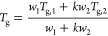
1where *w* is the weight fraction of each component and *k* is a model-specific constant depending on the densities and the expansivities of the polymers. The experimental results are in good agreement with that determined from the Gordon–Taylor equation, with a maximum deviation in *k* of 12% and a predicted *T*_g_ of 1.3 °C (see Tables S1 and S2), suggesting ideal mixing of the blend components.^36,37^ The trend in the *T*_g_ values of the HTPB blends is as follows: PER 5 wt % < GER 5 wt % < PER 10 wt % < GER 10 wt % < PER 15 wt % ≈ GER 13.57% < GER 15 wt %. Comparison between the two sets of blends with the different rosin esters within the same mass concentration shows that the *T*_g_ of the HTPB/GER blends is higher than that of the HTPB/PER blends, even though pure GER has a lower *T*_g_ than PER. The *T*_g_ of HTPB/PER(15 wt %) is comparable to that of HTPB/GER(13.57 wt %), the GER counterpart with equivalent molar concentration, so it appears that the effect of PER/GER addition on free volume and *T*_g_ depends on the number of rosin ester molecules instead of the mass (number of abietic groups),^38−40^ i.e., a PER molecule plays the same role as a GER molecule in terms of *T*_g_ modification despite its additional abietic functionality. Although the molecular mass of PER is higher than that of GER, there is a smaller quantity of PER in the blend within the same mass concentration, and this results in a lower blend *T*_g_ than that on inclusion of GER.

**Figure 3:**
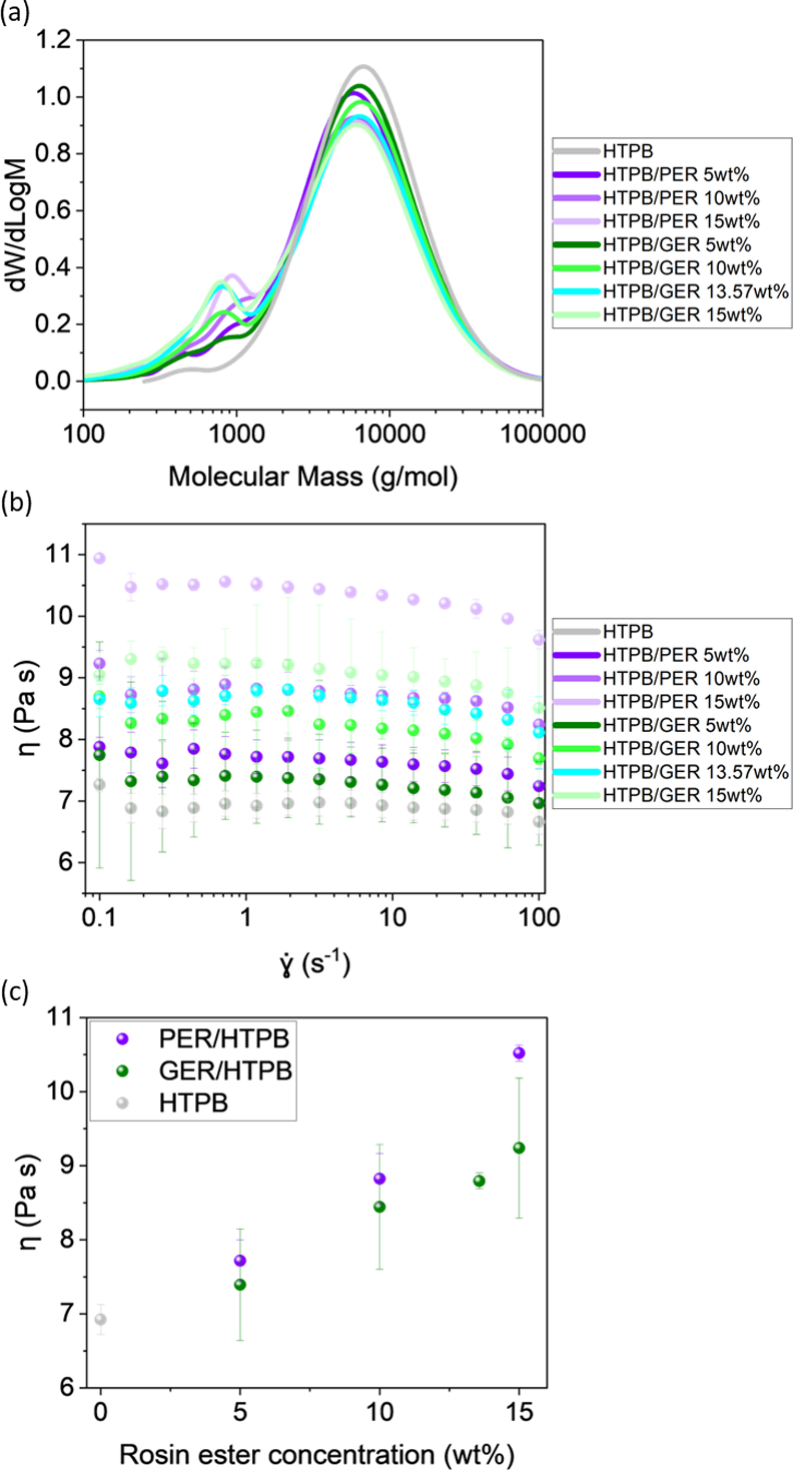
(a) GPC traces for HTPB/PER and HTPB/GER blends. (b) Plot of viscosity as a function of shear rate for the HTPB/PER and HTPB/GER blends at 25 °C. An increase in rosin ester concentration results in a more viscous blend, the relation of which is revealed in panel (c), which shows a plot of viscosity against rosin ester concentration at 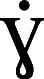 = 1.2 s^–1^.

Further evidence for the formation of homogeneous blends can also be provided from the GPC data in Figure 3a, where all GPC traces display two prominent peaks. The larger peak with a higher molecular mass at ∼6000 g/mol corresponds to HTPB, while the smaller peak with a lower molecular mass is associated with the rosin ester. While the larger HTPB peak position remains localized for both blends, the rosin ester peak position for HTPB/PER is significantly higher than that for HTPB/GER. With increasing rosin ester concentration, the rosin ester peak intensity increases, while the HTPB peak intensity decreases, demonstrating the effect of loading on the HTPB blends.

The modification of the viscosity behavior of the rosin esters can be revealed from the measurement of the viscosity of the blends (Figure 3b). The viscosity of neat uncured HTPB at 25 °C remains constant at ∼7 Pa s for all shear rates, from 0.1 to 100 s^–1^, in agreement with previously published values,^41−43^ a behavior typical of a Newtonian fluid. The incorporation of rosin ester leads to an increase in HTPB viscosity, the correlation of which is shown in Figure 3c. The viscosity of the HTPB blends follows in the order: GER 5 wt % < PER 5 wt % < GER 10 wt % < GER 13.57 wt % < PER 10 wt % < GER 15 wt % < PER 15 wt %. The viscosity of the PER blends is higher than that of the GER blends for the same mass concentration (and molar concentration) at all shear rates, suggesting stronger interaction between HTPB and PER due to the additional abietic group.^30^ This observation can be justified by the fact that pure PER has a higher viscosity than pure GER. It should be noted that with increasing rosin ester concentration, the drop in viscosity at a high strain rate is more prominent from ∼6 to ∼10%, indicating a shear thinning effect on addition of the rosin esters.

**Figure 4:**
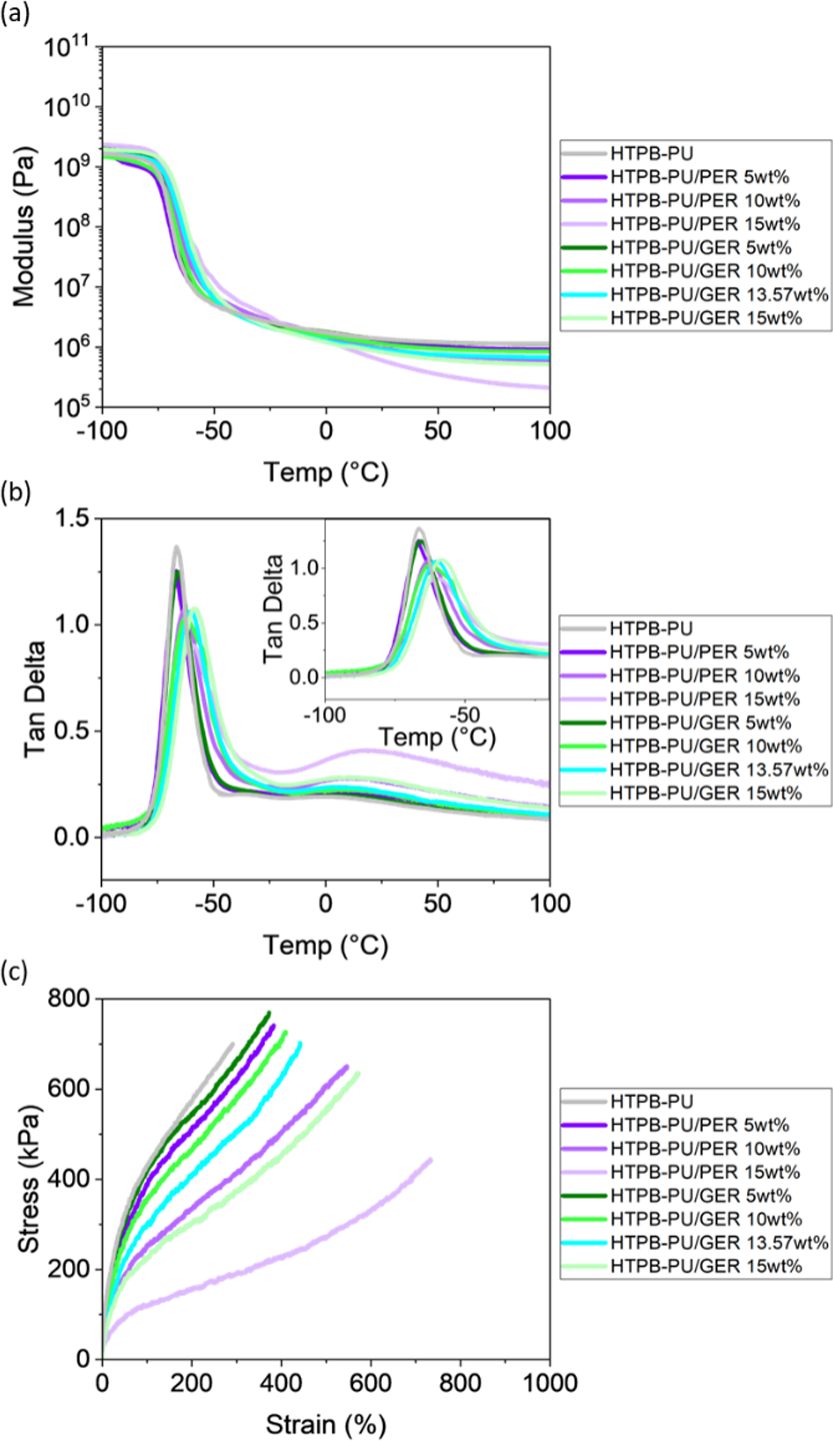
DMTA curves showing (a) storage modulus and (b) tan delta for HTPB-PU/PER and HTPB-PU/GER blends as a function of temperature and (c) representative stress–strain curves to failure for HTPB-PU/PER and HTPB-PU/GER blends.

HTPB blends are often cross-linked with isocyanates to form polyurethanes to tailor mechanical properties, such as elasticity, for a range of potential applications. Therefore, HTPB-based PUs with the same rosin ester concentrations were prepared (see Figure S3 for the corresponding FTIR spectra), and both the dynamic and quasi-static mechanical properties were measured. DMTA was performed to assess the change in elastic modulus and the corresponding tan delta behavior as a function of temperature (Figure 4). While all samples display comparable moduli, there is an abrupt decrease in the elastic modulus of up to 3 orders of magnitude at ∼ −65 °C where the glass transition process takes place. The values for the *T*_g_ were taken from the maximum in the tan delta (Figure 4b, inset) and are listed in Table 1. An increase in rosin ester concentration raises the *T*_g_ by a maximum of 8 °C compared to the HTPB-PU in the following order: PER 5 wt % < GER 5 wt % < PER 10 wt % < GER 10 wt % < PER 15 wt % ≈ GER 13.57% < GER 15 wt %. The trend is identical to the *T*_g_ distribution obtained for the uncross-linked HTPB blends. As discussed above, inclusion of GER has a greater effect on the shift in *T*_g_ than PER for the same mass concentration, which may be due to its higher molality.^38−40^

**Table 1:** Tensile Mechanical Properties of HTPB-PU/PER and HTPB-PU/GER

sample/wt % PER or GER	*T*_g_ (°C)	toughness (kPa)	Young’s modulus (kPa)	tensile strength (kPa)	elongation at break (%)	cross-link density (mol/m^3^)	free rosin ester (%)
HTPB-PU	–66.5	1059 ± 139	444 ± 15	722 ± 89	293 ± 68	59.0 ± 0.6	
HTPB-PU/PER(5)	–66.6	1506 ± 77	390 ± 10	778 ± 52	387 ± 30	49.5 ± 0.4	55
HTPB-PU/PER(10)	–62.9	1778 ± 147	344 ± 14	651 ± 89	546 ± 51	28.3 ± 0.5	44
HTPB-PU/PER(15)	–59.8	1580 ± 161	129 ± 14	427 ± 68	740 ± 94	7.7 ± 0.3	57
HTPB-PU/GER(5)	–65.6	1497 ± 201	418 ± 24	795 ± 105	377 ± 88	59.0 ± 0.2	31
HTPB-PU/GER(10)	–62.6	1468 ± 159	356 ± 33	724 ± 74	405 ± 78	47.0 ± 0.8	29
HTPB-PU/GER(13.57)	–59.8	1542 ± 179	299 ± 25	698 ± 76	442 ± 91	39.4 ± 0.2	32
HTPB-PU/GER(15)	–58.4	1867 ± 219	235 ± 18	644 ± 81	580 ± 115	26.9 ± 0.2	28

The tensile mechanical properties of these blends including tensile strength, tensile toughness, elongation at break, and Young’s modulus were determined, and the values obtained are listed in Table 1. Representative stress–strain curves to failure are also shown in Figure 4c. The incorporation of rosin ester leads to an improvement in the tensile toughness of the HTPB-based PUs, an increase in elongation at break of up to ∼400%, and the maximum stress to ∼800 kPa on inclusion of both rosin esters at 5 wt % loading. With increasing rosin ester concentration, the maximum elongation at break continues to increase, up to 740%, on addition of 15 wt % PER, while the tensile strength decreases before returning to that for HTPB-PU, to ∼430 kPa. The maximum decrease in Young’s modulus and tensile strength of HTPB-PU was −71 and −41%, but the increase in elongation at break and tensile toughness was +153 and +76% on inclusion of rosin ester. This behavior is similar to that reported previously,^26,30,38,44^ demonstrating the softening of HTPB (at RT) on inclusion of the rosin esters. Except for the 15 wt % GER blend (76%), the tensile toughness values of all rosin ester-containing HTPB-PUs are comparable, some 40 to 50% greater than that of HTPB-PU.

Given the comparable toughness values, the inclusion of the rosin esters to HTPB results in an increase in elongation at break but a reduction in tensile strength and Young’s modulus of HTPB in the following order: GER 5 wt % < PER 5 wt % < GER 10 wt % < GER 13.57 wt % < PER 10 wt % < GER 15 wt % < PER 15 wt %. This is a similar trend to that obtained for the viscosity results, confirming the viscoelastic modification of HTPB by the rosin esters, with PER having a greater effect than GER. To understand why such mechanical modification is obtained, the cross-link densities of the relevant PUs were determined from swelling tests in toluene (see SI4). The measured values, also listed in Table 1, show that there is a strong correlation between the cross-link density and mechanical properties, as expected, in that a lower cross-link density results in a lower Young’s modulus and tensile strength but a higher maximum elongation at break. The average molecular mass between cross-links (*M*_c_), determined from the DMTA plots in Figure 4a (Table S4), is also in good agreement with the cross-link density measurements in that the rosin ester-containing HTPB-PU with a lower *M*_c_ generally possesses a higher cross-link density. An increase in the number of cross-linking knots and entanglements hinders the movement of polymer chains, resulting in a stiffer material with a larger elastic modulus.^45−47^ However, in this work, inclusion of PER and GER results in a decrease in cross-link density. The abundance of hydroxyl groups in PER, confirmed from NMR and GPC analysis, may lead to side reactions with the isocyanate functional groups, in competition with the hydroxyl terminal groups of HTPB. This may allow the formation of urethane linkages with the rosin esters instead of the polymer chains, reducing the density of cross-links. As a result, the cross-link densities of HTPB-PU/PER are lower than that of HTPB-PU/GER for the same mass concentration, inducing a stronger softening effect on the HTPB-PU, but critically with an increase in tensile toughness and no major reduction in tensile strength.

**Figure 5:**
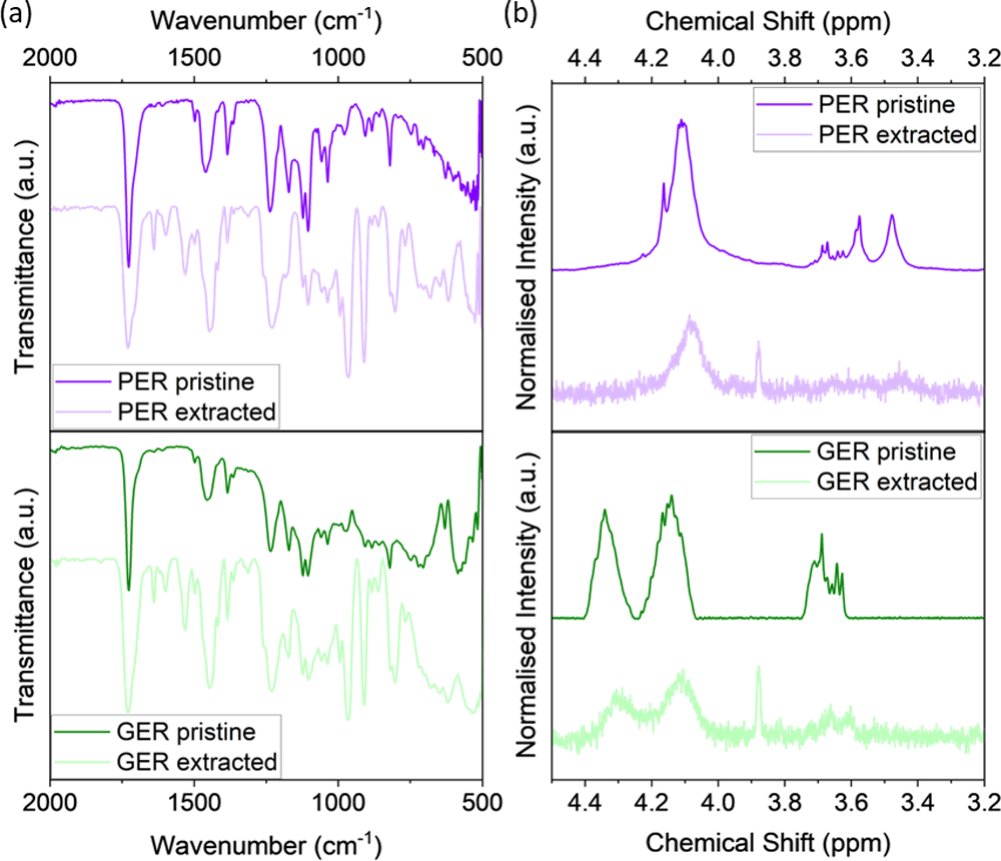
(a) FTIR spectra and (b) NMR spectra of materials extracted from HTPB-PU/PER 15 wt % and HTPB-PU/GER 15 wt % compared with pure PER and GER.

To understand the optimization of the current HTPB-based PUs, the amount of bound and free rosin ester was quantified post Soxhlet extraction of HTPB-PU/PER and HTPB-PU/GER with different rosin ester concentrations in toluene for 3 days. The extracted components were then examined by FTIR and NMR spectroscopy, where the respective spectra of HTPB-PU/PER 15 wt % and HTPB-PU/GER 15 wt % are shown in Figure 5 (spectra of samples with all rosin ester concentrations can be found in Figure S4). In the FTIR spectra (Figure 5a), the extracted materials display peaks comparable to those of the constituting neat rosin esters, with some additional peaks in the C=O region. The NMR spectra in Figure 5b also reveal similar characteristic peaks between the extracted materials and the corresponding rosin ester. A notable peak at ∼3.88 ppm can be observed for the extracted material, which is associated with the antioxidant (2,2′-methylene-bis(4-methyl-6-*tert*-butylphenol)) in HTPB. The data from both techniques suggest that the extracted materials are uncured rosin ester, and the proportions in each HTPB-based PU are listed in Table 1. Notably, for HTPB-PU/PER 15 wt % and HTPB-PU/GER 15 wt %, the proportion of uncured rosin was determined to be 57 and 28%, respectively, indicating a substantial amount of free rosin ester and further scope to tailor the mechanical properties of HTPB-PU.

## 4 Conclusions

The modification of the thermal and viscoelastic properties of HTPB blends on the inclusion of PER and GER was evaluated using a combination of physical and chemical characterization techniques. While the *T*_g_ of pure PER is higher than that of GER, the *T*_g_ of blends of HTPB and PER is lower than that obtained on inclusion of GER to HTPB before and after cross-linking with TDI. This behavior may be attributed to the equivalent role of free volume reduction by the rosin ester molecules. However, addition of PER had a more pronounced effect on the viscoelastic properties of HTPB, resulting in more viscous blends than HTPB/GER (prior to cross-linking) and producing a softer polyurethane with lower elastic modulus, slightly lower tensile strength, and higher maximum elongation. This behavior is consistent with the trends in the values obtained for the cross-link density of the blends. The abundance of hydroxyl groups in PER is in competition with the terminal hydroxyl groups of HTPB for reaction with the cross-linking agent TDI to form urethane bonds. This results in a lower cross-link density and more flexible HTPB. These results clearly demonstrate that addition of PER and GER to HTPB is an effective route for the modification of the mechanical and physical properties of HTPB blends and their related PUs, for use in solid propellant binders and coatings.

## Supporting Information Available

Chemical structures of HTPB, GER, and PER; GPC analysis; *T*_g_ determination using the Gordon–Taylor equation; FTIR spectra of HTPB, GER, PER, and blends; and measurement of cross-link density from swelling tests (PDF)
