# Supplementary material for: Pentaerythritol and Glycerol Ester-Based Rosin-Modified Hydroxyl-Terminated Polybutadiene (HTPB)
Source: ACS Polym Au. 2025 Jan 15;5(2):155–61. doi: 10.1021/acspolymersau.4c00089 (PMC11986720; doi:10.1021/acspolymersau.4c00089)
Supplement: Supplementary file 1 — lg4c00089_si_001.pdf [file lg4c00089_si_001.pdf]

Supporting Information

Pentaerythritol and Glycerol Ester based Rosin Modified Hydroxyl Terminated Polybutadiene (HTPB)

Frank Lee[1], Aran Guner[1], Ken Lewtas[1,2], Tony McNally[1*]

[1]International Institute for Nanocomposites Manufacturing (IINM), WMG, University of Warwick, UK

[2]The Falcon Project Ltd., Astley, Manchester, M29 7NW, UK

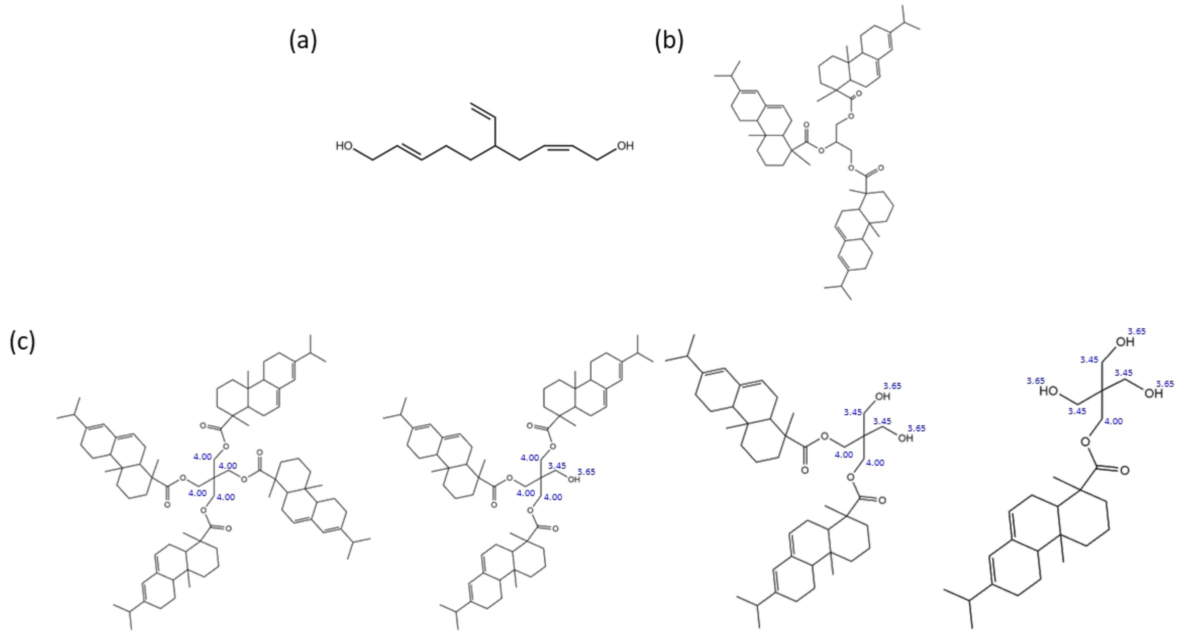

Fig. S1 Chemical structures of a) HTPB, b) GER and c) PER showing from left to right the tetra-, tri-, di- and monoester and their corresponding ^{1}H NMR simulated chemical shifts (ChemDraw Ultra, version 11.0). Only the chemical shifts between 3.3 and 5.5 ppm are shown.

As discussed with regards Fig. 1 b), the intense peak at 4.11 ppm matches the simulated chemical shift for -CH_2-O from the ester while peaks at 3.48, 3.58, 3.67 ppm match the simulated chemical shift of -CH_2-OH from the alcohol as depicted in Fig. S1 c). It is worth noting that the chemical shifts of the ester or alcohol (if any) are the same among tetra-, tri-, di- and monoester, hence allowing calculation of the esters composition as follows:

Let the area of the ester peak, contributed by 2 protons per ester group, at 4.11 ppm be A_{Ester}, and the area of alcohol peaks, contributed by 3 protons per hydroxyl group, at 3.48, 3.58, 3.67 ppm be $A_{Alcohol}$. As the chemical shifts are independent of the degree of esterification, a reasonable and trivial assumption of the mole ratio of 0:1:3 for mono-, bi-, and tri- abietic ester will be made, and let the mole fraction of the bi-ester be x, the mole fraction of tri-ester be $3x$, where x=0.131. Then,

$$\frac{A_{Ester}/2}{A_{Alcohol}/3} = \frac{x \cdot 2 + 3x \cdot 3 + (1 - x - 3x) \cdot 4}{x \cdot 2 + 3x \cdot 1} \quad (1)$$

Hence, the mole fractions of tetra-, tri-, di- and monoester are 47.2%, 39.6%, 13.1%, and 0%, respectively.

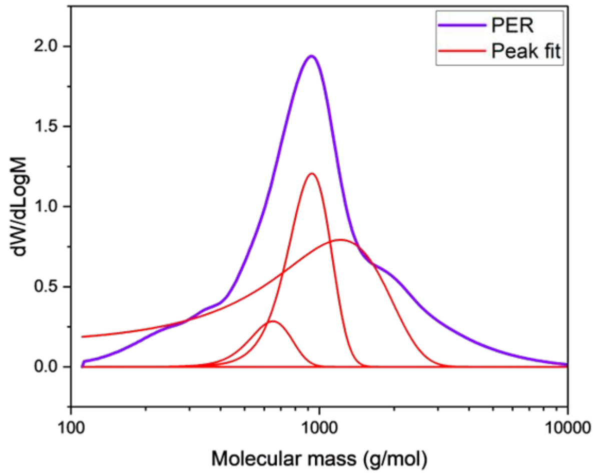

Fig. S2 Gaussian peak fit for GPC trace of PER. Peaks are selected at 364, 650, 933, and 1217 g/mol for the mono-, di-, tri-, and tetra-ester, respectively. GPC is calibrated by poly(methyl methacrylate) standards. Note that the fitting of the 364 g/mol peak is almost flat, suggesting a very low monoester content.

Assuming dW/dLogM depends only on the number of atoms (abietic groups), the area of each peak fit represents the mass concentration of different esters, *i.e.*,

$$Area\ ratio = molecular\ mass \times mole\ fraction \quad (2)$$

The mole fractions of each ester can then be computed by dividing the area ratio by their corresponding molecular mass, which are found to be 55%, 30%, 15%, and 0% respectively for of tetra-, tri, di-, and monoester. The lower molecular weight components cannot be accurately fitted using the usual distributions (*i.e.*, Gaussian) because the material is steam stripped to remove the light ends. However, the estimates of the concentrations of the components are in reasonable agreement with the NMR data.

Table S1 Parameters used in the application of the Gordon-Taylor equation.

T_g of HTPB ($T_{g,1}$)	-79.1°C
T_g of PER ($T_{g,2}$)	53.1°C
T_g of GER ($T_{g,2}$)	42.4°C

Table S2 T_g determined experimentally and that predicted using the Gordon-Taylor equation, taking the k value for 5wt%.

k determination			Experimental T_g			Predicted T_g		
w_2	PER/HTPB	GER/HTPB	w_2	PER/HTPB	GER/HTPB	w_2	PER/HTPB	GER/HTPB
5%	0.292	0.350	5%	-77.1	-76.9	5%	-77.1	-76.9
10%	0.274	0.338	10%	-75.2	-74.7	10%	-74.9	-74.5
13.57%		0.296	13.57%		-73.7	13.57%		-72.8
15%	0.227	0.289	15%	-74.0	-73.2	15%	-72.6	-72.0

The parameters in the Gordon-Taylor equation, $T_g = \frac{w_1 T_{g,1} + k w_2 T_{g,2}}{w_1 + k w_2}$ are listed in Table 1 ($T_{g,1}$, $T_{g,2}$) and Table 2 (w_2), while $w_1 = 1 - w_2$. Using these values together with the experimentally measured T_g (columns 4-6), k can be determined for every weight percentage of PER and GER added to HTPB (columns 1-3). The error in k (standard deviation divided by mean) is found to be less than 12% for both blends. One can also extrapolate the T_g using the value of k computed for 5wt%, and the predicted T_g for the blends with 10wt%, 13.57wt%, and 15wt% rosin ester are listed in columns 7-9. The deviation of the predicted value from the experimental value is found to be less than 1.3°C for all blends.

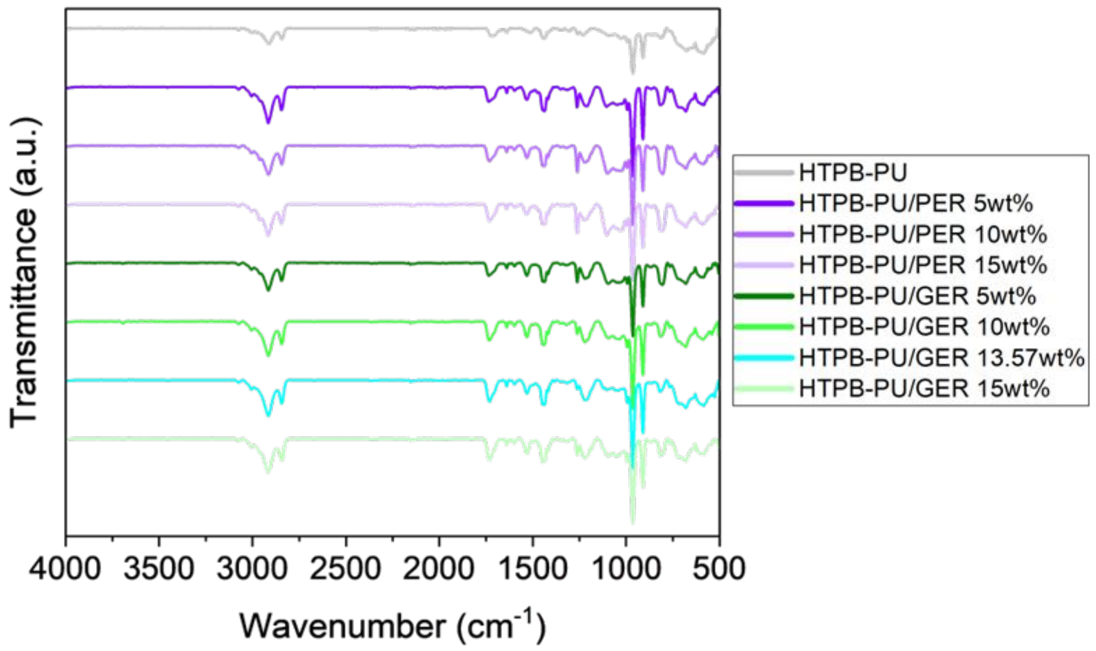

Fig. S3 FTIR spectra of HTPB-PUs.

Supporting Information S4 Measurement of crosslink density from swelling tests

Samples were immersed in toluene until steady mass is reached, which was measured as the swollen mass m_s. After drying, the samples were weighed again as the de-swollen mass m_{ds}, and the volume fraction of the polymer in the swollen specimen v calculated from,

$$v = \frac{m_{ds}/\rho_p}{m_{ds}/\rho_p + (m_s - m_{ds})/\rho_s} \quad (3)$$

where, ρ_p and ρ_s are the density of the polymer and the solvent, respectively.

The crosslink density n can then be determined from,

$$n = -\frac{\ln(1-v) + v + \chi v^2}{V_s\left(v^{\frac{1}{3}} - \frac{v}{2}\right)} \quad (4)$$

where, χ is the polymer-solvent interaction parameter ($\chi = 0.36$ for HTPB-toluene system) and V_s is the molar volume of the solvent ($V_s = 106.2\ cm^3/mol$ for toluene).

Table S4 Average molecular mass between crosslinks from DMTA

**Average molecular mass between crosslinks (g/mol)**		
Rosin ester concentration	HTPB-PU/PER	HTPB-PU/GER
5%	2061	1797
10%	2603	2192
13.57%		2696
15%	4415	3027

The molecular mass between crosslink M_c can be determined from,

$$M_c = \frac{\rho_p RT}{G} \quad (5)$$

where, G is the modulus at rubbery plateau measured by DMTA, ρ_p is the density of the polymer, R is the gas constant and T is the temperature.

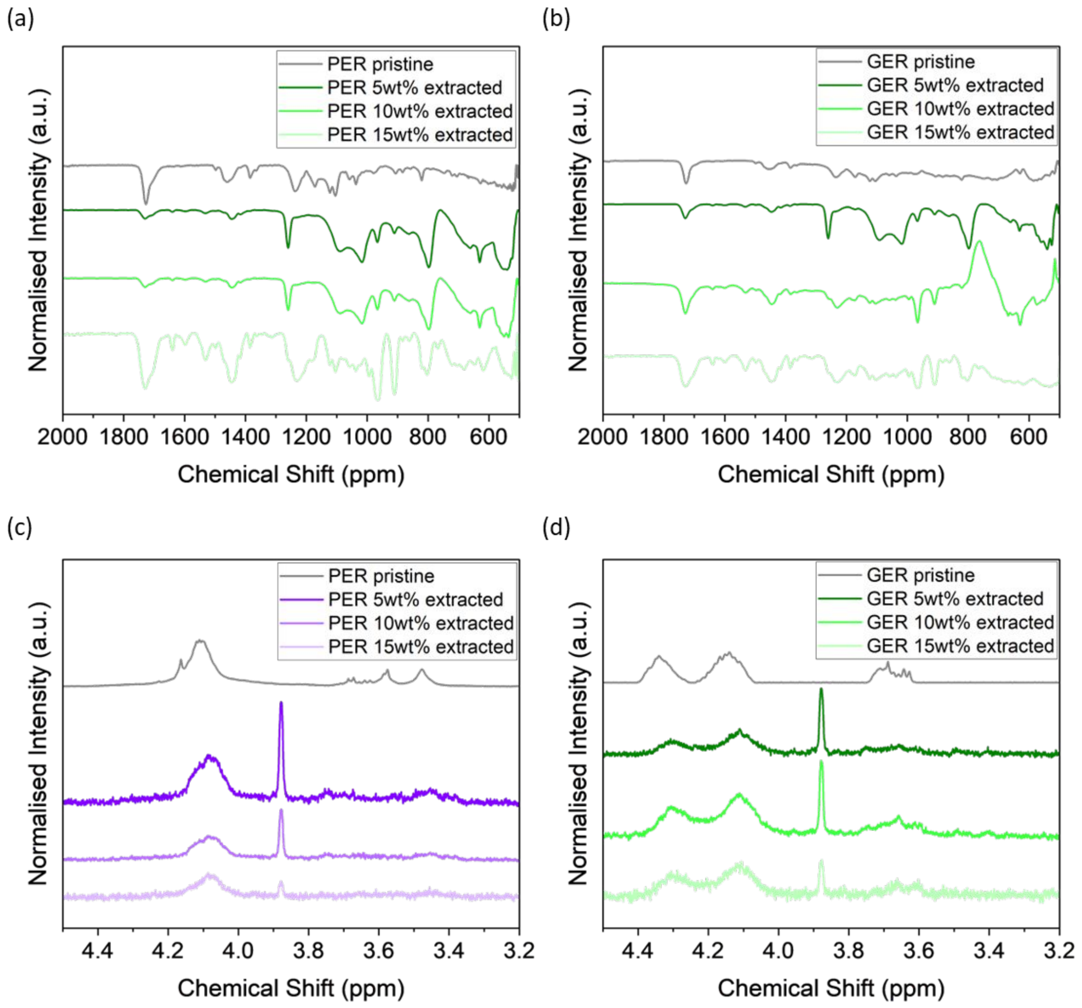

Fig. S4 a) FTIR a) and b) and NMR c) and d) spectra of the materials extracted from HTPB-PU/PER and HTPB-PU/GER with different rosin ester concentrations.
